# Editorial Comment to Prostate squamous cell carcinoma developing 11 years after external radiotherapy for prostate adenocarcinoma

**DOI:** 10.1002/iju5.12171

**Published:** 2020-06-01

**Authors:** Sho Uehara

**Affiliations:** ^1^ Department of Urology Tokyo Medical and Dental University Tokyo Japan

Matsugasumi *et al*. experienced a prostate squamous cell carcinoma which developed 11 years after radiotherapy.^1^ Zelefsky *et al*. reported that the 10‐year likelihoods for bladder or colorectal cancer development in brachytherapy were 2%.^2^ In recent guidelines, the follow‐up method after radiotherapy mainly depends on serum prostate‐specific antigen testing, and routine checks for secondary malignancy after treatment are not considered.^3^ A few studies have reported that secondary malignancy may increase over time, however.^4^ The potential risk of a late increase in secondary malignancy has not been evaluated with a sufficient follow‐up period. Reports show that the current cancer death rate, including prostate cancer, continues to decline.^5^ Given the prolonged survival time after treatment, follow‐up methods should be considered in the long run. In this report, the patient suffered urination difficulties including hesitancy and pain. Regular interviews after treatment could provide clues to the diagnosis of secondary malignancy. Whether follow‐up should be discontinued if prostate‐specific antigen remains stable or whether imaging studies should be performed regularly to rule out secondary malignancy remains unclear. To establish an optimal follow‐up method, further study with a longer follow‐up period is warranted.

## Conflict of interest

The author declares no conflict of interest.
